# Wideband 1-bit reconfigurable transmission metasurface unit cell design in Ka-band with polarization hold and conversion

**DOI:** 10.1038/s41598-023-47466-1

**Published:** 2023-11-16

**Authors:** Tongxing Huang, Wenjie Fu, Dun Lu, Yibo Pan, Minxing Wang, Yang Yan

**Affiliations:** Terahertz Science and Technology Key Laboratory of Sichuan Province, School of Electronic Science and Engineering, University of Electronic Science and Technology of China, Chengdu, 610054 China

**Keywords:** Electrical and electronic engineering, Electronic and spintronic devices

## Abstract

In this paper, a wideband transmission unit cell is proposed for programmable metasurfaces operating in the Ka-band. The unit cell features a compact period of only 2.91 mm, corresponding to 0.34 *λ*_0_ at the center frequency of 35 GHz. A receiving layer, consisting of a patch loaded with two PIN diodes, is utilized to achieve 1-bit phase modulation, while a U-shaped patch serves as the transmitting layer to enable selection of linear polarization hold or conversion. Based on the multi-resonance principle, the proposed unit cell exhibits broadband behavior, as demonstrated by simulation results under periodic boundary conditions, which indicate a 3 dB transmission bandwidth of 29.4–40 GHz (30.5%). Two unit cells were fabricated and tested in a standard waveguide, with the minimum insertion loss of the two states tested being 1.2 dB and 3 dB bandwidths of 30.1–31.2 GHz and 33.5–38.5 GHz, respectively. The maximum 180° phase error is 10°, indicating the high quality of the proposed unit cell.

## Introduction

Metasurfaces have demonstrated remarkable proficiency in manipulating the amplitude, phase, and polarization of electromagnetic waves, and garnered considerable interest in a variety of fields, including satellite communication, microwave and millimeter wave radar, and high-resolution imaging systems^1–5^. The programmable metasurface (PM) has further augmented the potential and opportunities of metasurfaces, propelling their utility to a new echelon^6^. The unit-cell of a PM is commonly composed of integrated electronic-controlled components, including positive-intrinsic-negative (PIN) diodes^7, 8^, varactors^9, 10^, and micro-electro-mechanical systems (MEMS)^11^. PIN diodes have garnered significant attention in research due to their highly advantageous programmable digital control capability, as well as their ability to operate over a wide bandwidth. In the context of PM, transmit arrays exhibit superior characteristics over reflect arrays due to the absence of feeder blocking effects.

A typical transmissive PM unit comprises two microstrip patch layers, namely the receiver (RX) and the transmitter (TX). The RX layer is responsible for receiving the incident wave and converting it into a guided wave signal. This guided wave signal is then coupled to the TX layer, where it is radiated into free space. In the present methodology, a patch or phase shifter equipped with an integrated PIN diode can efficiently modulate the phase in a digital manner. A PM transmissive array was proposed in reference^12^. The RX is responsible for generating the induced currents that are subsequently conducted by the central metal vias of the unit cell to the TX. The TX unit incorporates two PIN diodes that radiate electromagnetic waves to free space in 1-bit discrete phase. In a similar vein, the unit cell proposed in reference^13^ incorporates two pin diodes within both the RX and TX layers to enable 1-bit phase modulation.

In recent years, there has been a growing interest among researchers to broaden the operating bandwidth of transmissive PM arrays, and various approaches have been explored^14^. The air-gap design proposed in^15^ achieves broadband polarization conversion from 4 to 6.5 GHz by utilizing air as a low-permittivity dielectric layer. However, air-coupled structures can be difficult to fabricate and integrate into metasurface devices because maintaining uniform air gap thicknesses requires precise mechanical assembly or sacrificial layer techniques. And air gaps leave metasurface layers suspended, making the overall structure fragile and prone to damage while also preventing standard monolithic integration approaches like stacking layers. The multi-layer transmissive PM unit cell described in^16^ employs a 7-layer printed circuit board (PCB) obtains a broadband effect with a 3 dB transmission bandwidth of 32% around 12.5 GHz. However, multilayer PCBs increase complexity through more complex design and fabrication requirements. Thus, these approaches may not be suitable for components with higher frequencies and finer dimensions. Alternative approaches, such as conducting induced currents by metal posts, have been explored but have exhibited significant deficiencies in operating bandwidth, particularly at millimeter wave frequencies where the impact of microstrip lines and lumped elements on operating bandwidth and transmittance efficiency is pronounced^17^.

This paper investigates the expansion of the unit cell bandwidth based on multi-resonance theory and presents a compact wide-band reconfigurable 1-bit transmissive PM unit cell in Ka-band, offering two distinct solutions for the purpose of polarization holding and polarization conversion. The unit cell comprises of an innovative RX patch and slotted TX patch antenna, linked with a metal through-hole located at the center. Simulation results demonstrate that the proposed unit cell has a 3 dB transmission bandwidth of up to 30.5%. Further, a 1×2 array was designed for waveguide testing, yielding promising results in experimental analysis.

## Unit cell design and operation principle

**Figure 1:**
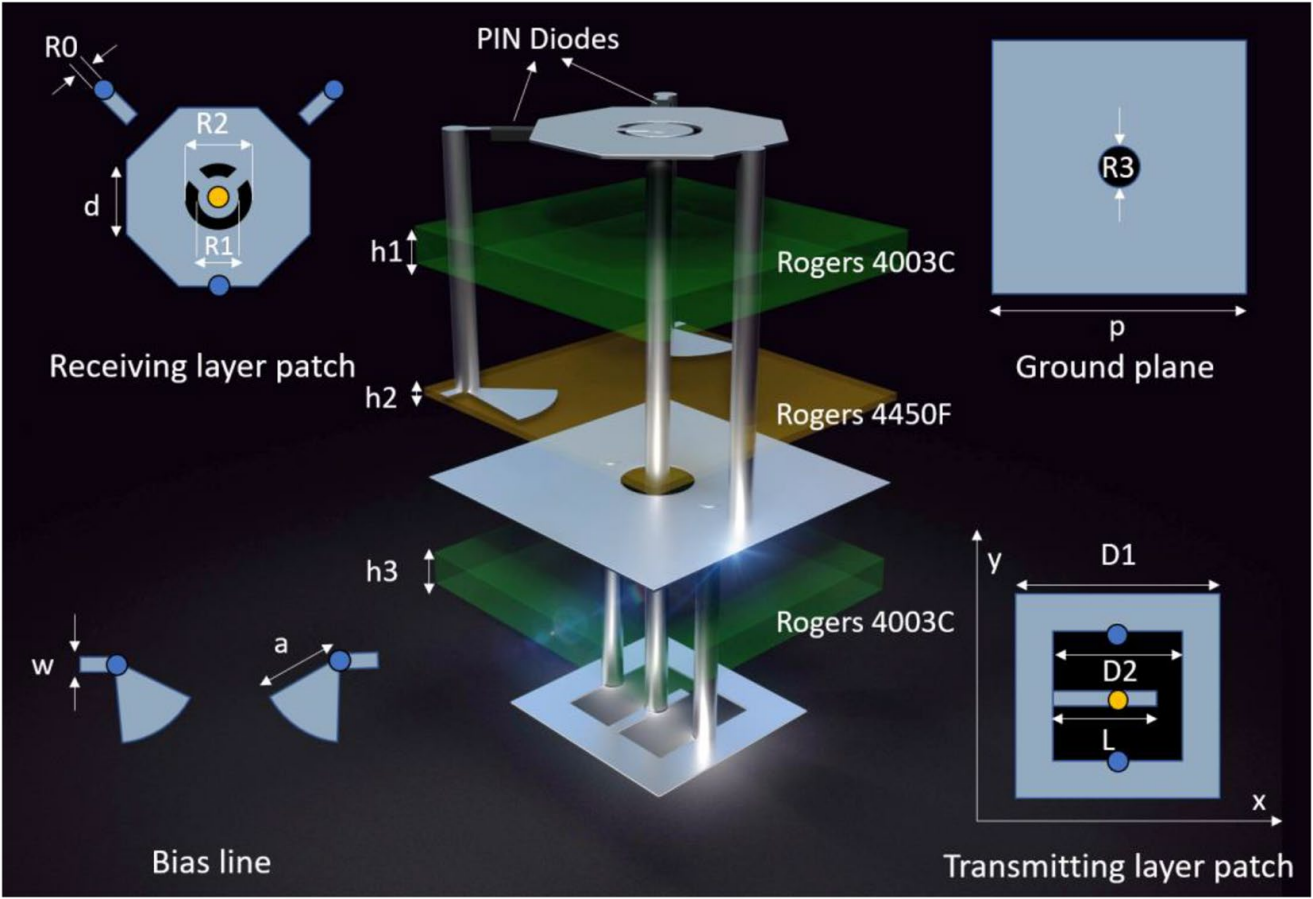
Geometry of the proposed 1-bit PM unit cell.

The present study introduces a novel 1-bit PM unit cell structure, as depicted in Fig. 1, that comprises three dielectric layers and four metal layers with dimensions of 2.91 mm × 2.91 mm. The receiving and transmitting layers are fabricated on two Rogers 4003C substrates having a uniform thickness of *h*_1_ = *h*_3_ = 0.503 mm. The intermediate layer, fabricated using a Rogers 4450F substrate with a thickness of *h*_2_ = 0.1mm, acts as a connecting layer between the two aforementioned layers. The bias and ground layers are printed on both sides of the structure. The receiver is an octagonal patch having a square shape with a centrally scooped-out ring, which is interconnected to the transmitter through a metallized via-hole. Furthermore, the internal circular patch and octagon are conjoined to form a fundamental loop. The receiver incorporates two inverted pin diodes symmetrically located at the corners and interconnected to the bias line via metallized blind holes. Another metallized blind hole is introduced at the lowest point of the receiver's electric field to establish a connection with the ground. The transmitting layer comprises a U-shaped patch with grounded metal holes that function as a polarization selector. The U-shaped patch is oriented along the x-axis to transmit x-polarized waves and along the y-axis to transmit y-polarized waves. The DC supply is connected to bias lines, and two fan stubs are incorporated to minimize insertion loss and improve DC/RF isolation. The optimization of all relevant parameters has been presented in Table 1.

**Table 1:** Parameters of the unit cell.

Symbol	Value (mm)	Symbol	Value (mm)
*h*_*1*_	0.508	*d*	0.83
*h*_*2*_	0.1	*w*	0.1
*h*_*3*_	0.508	*a*	0.8
*R*_*0*_	0.2	*p*	2.91
*R*_*1*_	0.6	*D*_1_	1.8
*R*_*2*_	0.8	*D*_2_	1.2
*R*_*3*_	0.5	*L*	0.9

**Figure 2:**
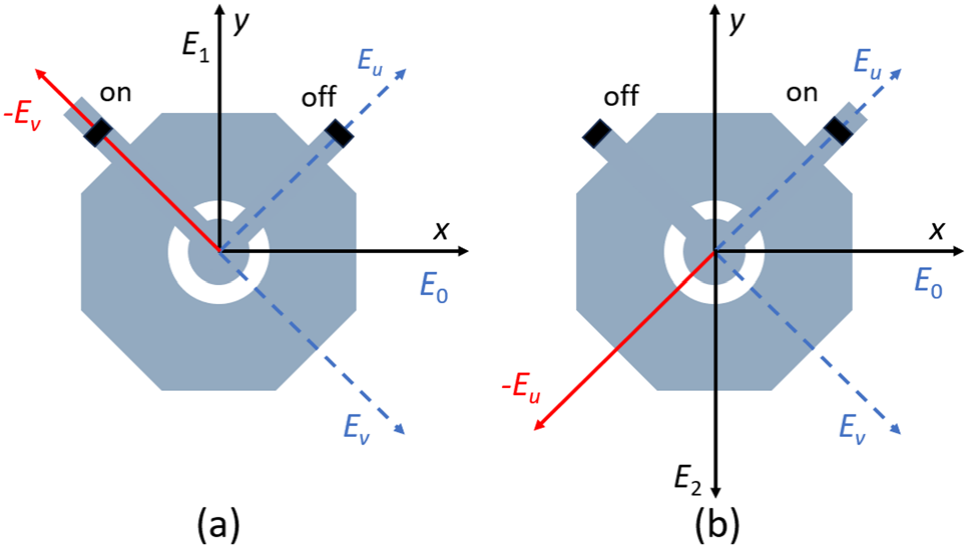
180° phase shift operation of the receiving patch: (**a**) state 1 and (**b**) state 2.

The PIN diode utilized in this study is the Macom MA4GP907 model. Its on-state characteristics can be represented by a series combination of a 4.2 Ω resistor and a 0.05 nH inductor, whereas its off-state characteristics can be represented by a parallel combination of a 300 kΩ resistor and a 42 fF capacitor. The PIN diode exhibits two distinct operating states, denoted as states 1 and 2, which correspond to the on and off states or the off and on states, respectively. These states are responsible for modifying the length of the phase delay line located on the octagon. The present study aims to elucidate the decomposition of the incident wave electric field ${E}_{0}$ polarized in the *x* direction into two sub-fields in the *u* and *v* directions, where ${E}_{0}=\frac{{E}_{u}}{\sqrt{2}}+\frac{{E}_{v}}{\sqrt{2}}$. Specifically, when the PIN diode operates in the state 1, as illustrated in Fig. 2(a), the PIN-diode on the left is activated while the diode on the right is deactivated, which is equivalent to increasing the delay line length on the left while leaving the right unchanged. the alteration of the delay line is anticipated to augment the phase shift in the *v* direction by 180°. Henceforth, the novel synthetic domain may be represented as follows,

1 $${E}_{1}={E}_{u}/\sqrt{2}-{E}_{v}/\sqrt{2}={E}_{y}$$

Similarly, in the event that the PIN diode is functioning in the state 2, as illustrated in Fig. 2b, the composite field may be delineated as follows:

2 $${E}_{2}=-{E}_{u}/\sqrt{2}+{E}_{v}/\sqrt{2}=-{E}_{y}$$

From a theoretical perspective, it can be postulated that ${E}_{1}$ and ${E}_{2}$ exhibit a phase difference of 180°.

To illustrate the operation principle and evaluate the performance of the proposed unit cell, the CST full-wave simulation in periodic boundary conditions (PBC) as infinite array environment are employed. The incident wave is polarized normal incidence along the *x*-axis direction to the upper surface of the unit cell. In this paper, the incident wave polarized along the *x*-axis is defined as ${E}_{xi}$. Likewise, the polarization of the transmitted wave along the x-axis is defined as ${E}_{xt}$ and along the *y*-axis as ${E}_{yt}$. Thus, the co-polarization transmission coefficient can be expressed as

3 $${T}_{xx}={E}_{xt}/{E}_{xi}.$$

and the cross-polarization transmission coefficients can be expressed as

4 $${T}_{xy}={E}_{yt}/{E}_{xi}$$

**Figure 3:**
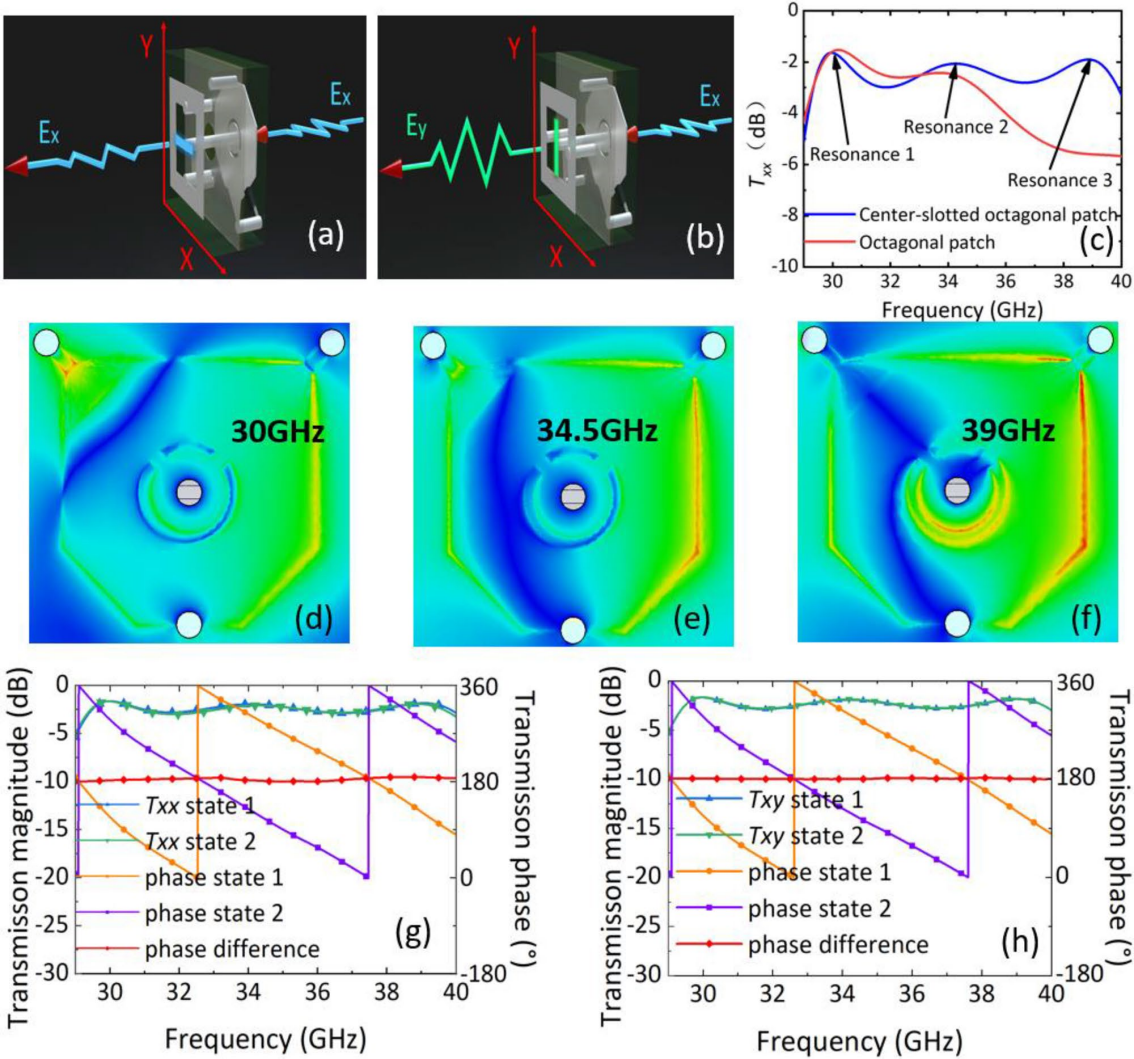
(**a**) Scheme of co-polarization transmission for U-shaped patch pointing to *x*-axis, (**b**) scheme of cross-polarization transmission for U-shaped patch pointing to *y*-axis. (**c**) The *T*_*xx*_ transmission characteristics for the octagonal patch and the center-slotted octagonal patch in the state 1. The electric field distribution excited at (**d**) 30 GHz, (**e**) 34.5 GHz and (**f**) 39 GHz. (**g**) Simulation results of co-polarization transmission coefficient and phase of two states. (**h**) Simulation results of cross-polarization transmission and phase of two states.

As shown in Fig. 3a, b, the orientation of the U-shaped patch can be oriented in the x or y direction to transmit the x-polarization and y-polarization, respectively. While the incident wave is constantly x-polarized, and thus the two orientations will function as co-polarized and cross-polarized transmissions, respectively. The bandwidth characteristics of the unit cell are analyzed in a co-polarized transmission mode. The Txx transmission characteristics for the octagonal patch and the center-slotted octagonal patch in the state 1 are shown in Fig. 3c, corresponding to the dual- and tri-resonant modes of operation, respectively. For octagonal patch, only the resonances at 30 GHz and 34.5 GHz can be excited. By introducing the centrally scooped-out ring, an additional resonance at 39 GHz would be excited. This denotes that in the proposed receiver design, the square octagonal patch serves as a dual resonator with two resonant frequencies, while the circular patch introduces an additional resonant frequency. The electric field distributions excited at 30, 34.5 and 39 GHz in the state 1 are presented in Fig. 3d–f, which shown that an additional resonance mode could be generated at 39 GHz other than the resonance modes at 30 and 34.5 GHz. This additional resonance could extend the operating bandwidth to higher frequencies.

Figure 3g shows that the two states exhibit co-polarization transmission coefficients that are well-matched, with a 3 dB bandwidth of 29.4–40 GHz (30.5%). The lowest insertion loss is observed at 29.9 and 30 GHz for states 1 and 2, respectively, with values of 1.64 dB. In this case, it was observed that the asymmetry in the symmetry axes of the top and bottom layers of the unit cell leads to a slight variation in the co-polarization transmission coefficient. Furthermore, the 180° phase difference in the transmitted field between the two states remains highly stable across the operating bandwidth due to the distinct operating states of the PIN diode resulting in current reversal. The maximum phase error observed was 8.7° at 38.5 GHz , which is within acceptable limits.

As depicted in Fig. 3h, the cross-polarization transmission of the two states evinces complete coherence when the U-shaped patch is oriented in the y-axis direction. This coherence arises from the y-axis symmetry of the cell's front and back sides. The 3 dB bandwidth spans 29.4–40 GHz (30.5%), while the minimum insertion loss is 1.64 dB at 30 GHz. Meanwhile, it is observed that the superior symmetry of the proposed unit cell leads to a more stable phase difference with a maximum phase error of merely 2.5° at 35.8 GHz between the two states. This indicates that the unit cell is proficient in generating a steady 1-bit phase difference across a broad bandwidth and in selecting the desired polarization of the transmission field. It is worth noting that the phase flip arises from the PIN diode state reversing the current flow direction along the delay line. This reversed current occurs at only one point on the delay line, altering the unit cell current in the plane of symmetry. Since the designed unit cell exhibits good symmetry, Txx and Txy remain nearly identical for both states. In the latter half of this thesis, to facilitate waveguide simulations, the unit cells are employed in the configuration of U-shaped patches oriented in the x-axis direction, resulting in co-polarization of all transmission coefficients.

## Waveguide simulation and testing

**Figure 4:**
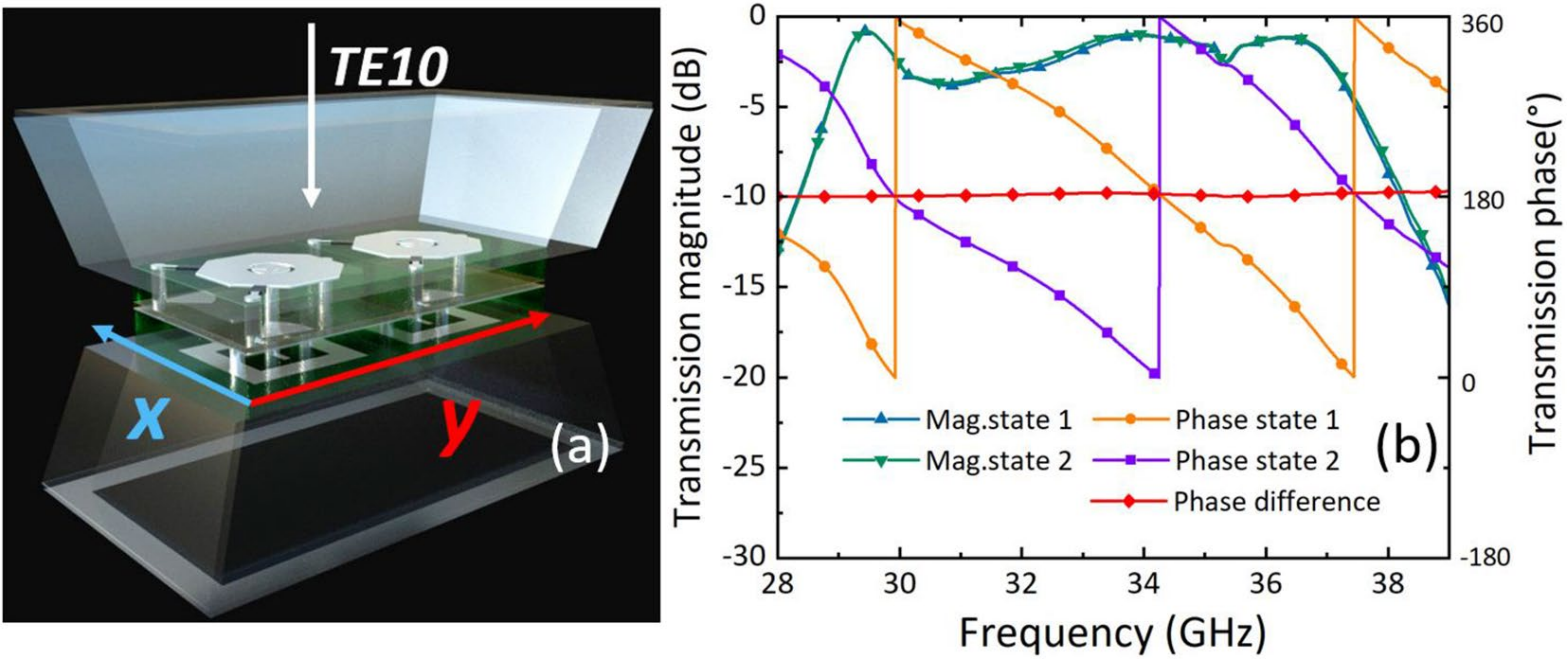
Waveguide simulation of two states under two unit cells arrangement: (**a**) two unit cells arranged along the y-axis and bounded by electric field perpendicular to the y-axis and magnetic field perpendicular to the x-axis (**b**) the transmission coefficients and phase of two states.

To validate the 1-bit phase modulation and polarization control functionality of the proposed unit cell, simulations were conducted with a standard rectangular waveguide to model plane wave illumination of an infinite periodic array^19^. The proposed unit cell features substantially reduced dimensions (2.91 × 2.91 mm) when compared to the established Ka-band standard waveguide, namely WR-28 (3.556 × 7.112 mm). To circumvent the waveguide transmission mismatch stemming from the highly discrepant dimensions, the waveguide simulation is subjected to simulations and experiments, wherein two unit cells are placed in an adjacent manner. The primary mode TE10 of the rectangular waveguide exhibits electric field orientation that is perpendicular to the wide edge of the waveguide. Consequently, two unit cells are arranged along the y-axis. To enable characterization with rectangular waveguide ports and facilitate experimental measurements, the orientation of the U-shaped patches was aligned to the x-axis, ensuring co-polarized transmission for both the waveguide simulations and laboratory tests, as depicted in Fig. 4a. In order to simulate the real measurement situation and to ensure smooth matching of the TE10 mode to the cell, two rectangular transition waveguides with a thickness of 2 mm were designed to minimize impedance mismatch. The PIN diode switching states of both unit cells are concurrently set to either state 1 or state 2, whereby a theoretical maintenance of a 180° phase difference between the two states is achieved.

Figure 4b presents the simulated transmission coefficients and phase of two states, which demonstrate the multi-resonant mode operation of the unit cells at resonant frequencies of approximately 30, 34, and 37 GHz. The two states of S21 exhibit a high degree of correspondence, with a 3 dB bandwidth of 29.0–37.2 GHz. The waveguide inherently introduces dispersion, meaning different frequencies propagate at slightly different velocities through the waveguide medium. The waveguide dispersion properties lead to the observed reduction in bandwidth and shift to lower frequencies relative to the periodic boundary condition simulations. This dispersion causes a distortion and shift in the frequency response compared to free space simulations. The degradation of the transmission coefficient from 30 to 32 GHz can be attributed to the oblique incidence of the TE10 mode in the rectangular waveguide, which exacerbates the dispersion effects. Notably, the expected 180° phase difference between the two states is well-preserved except at the locations of distortion, where a maximum error of 4° at 33 GHz is observed.

**Figure 5:**
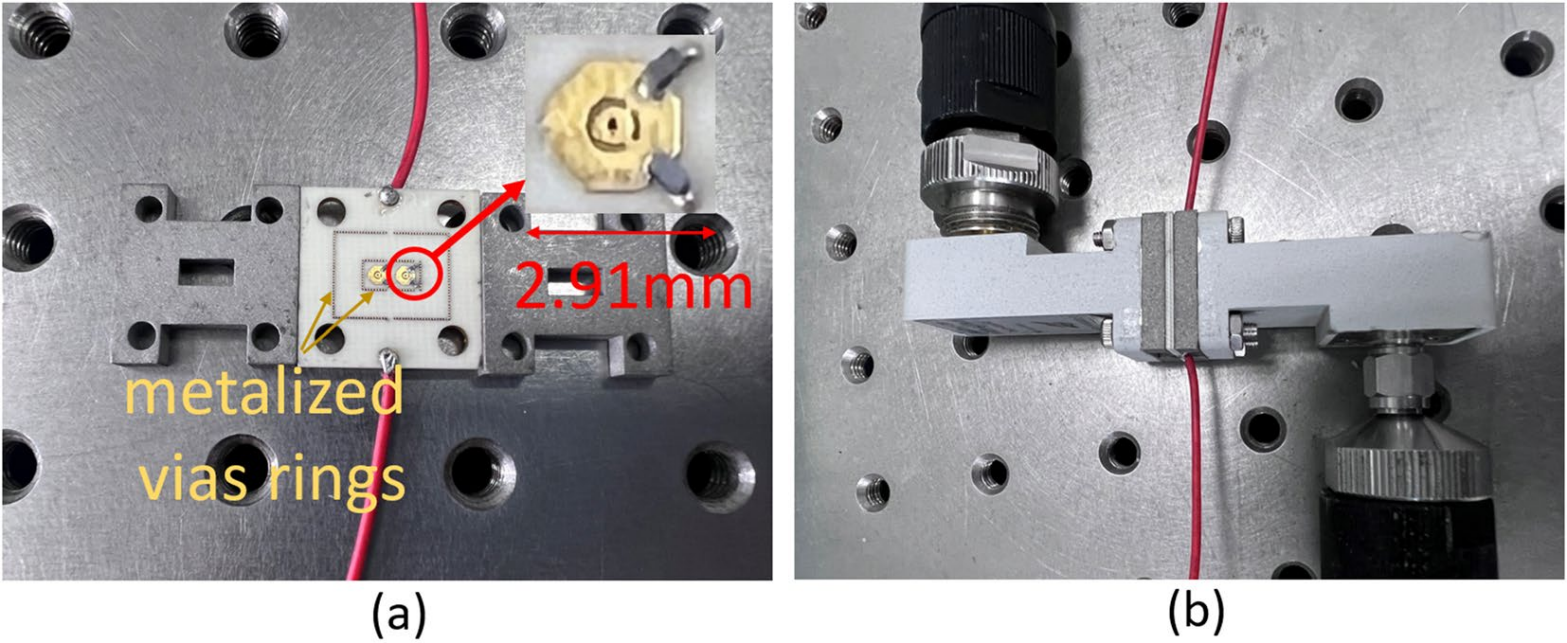
Photos of a fabricated prototype: (**a**) unit cells and waveguide transition section (**b**) the measurement system.

To experimentally validate the proposed design, the two rectangular transition waveguides and unit cells were fabricated and measured. The fabricated parts were specifically designed with metalized vias rings around two cells arranged along the y-axis to ensure continuity of the waveguide walls and limit the elements to a size of 2.91 × 5.82 mm to avoid propagation of substrate modes outside the unit cells. Two extended wires were connected in parallel to the DC power to control 4 PIN diodes simultaneously and obtain two phase states. The waveguides were connected to the two coax-to-WR28 adaptors at both ends and then to the two ports of the vector network analyzer. The fabricated unit cells and experimental setup are shown in Fig. 5a, b, respectively.

**Figure 6:**
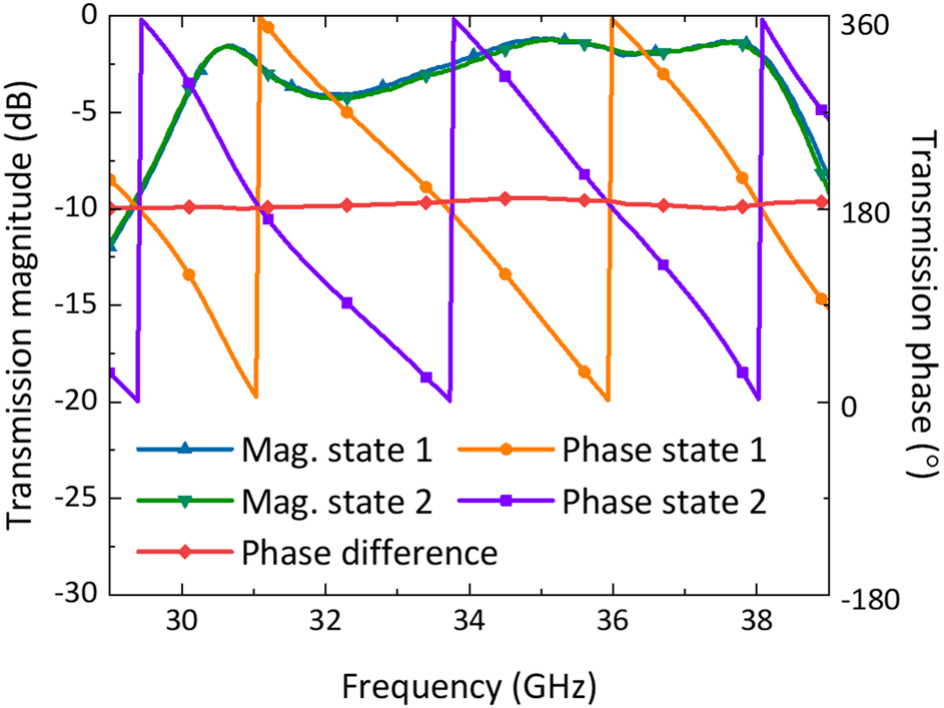
The measured transmission coefficients and phase of the two states.

The results of this study demonstrate the efficacy of a 1-bit broadband transmissive unit cell based on multi-resonant mode. Figure 6 displays the measured co-polarized transmission coefficients and phase difference between two states, which show perfect agreement and operate in multi-resonant mode with three resonances at approximately 30.6, 35, and 37.8 GHz. The operating 3 dB bandwidth spans 30.1–31.2 GHz and 33.5–38.5 GHz, with a 3 dB drop in the transmission coefficient occurring from 31.2 to 33.5 GHz. Manufacturing tolerances may have caused variations in the delay line length at the PIN diode, leading to some amplitude deterioration. State 1 exhibits a measured minimum insertion loss of 1.2 dB at 35.2 GHz, while state 2 experiences 1.2 dB at 35.4 GHz. Additionally, the phase difference between the two states maintains a good 180° phase difference and experiences a maximum error of 10° at 34.8 GHz. The measured transmission coefficient and maximum phase error exhibit a slight downward frequency shift compared to simulations. This could arise from the small unit cell size or other mechanical tolerances in the experimental setup. Overall, these findings confirm the effectiveness of the 1-bit broadband transmission PM unit cell based on multi-resonant mode.

## Conclusion

A broadband 1-bit transmissive PM unit cell, operating in the Ka-band, is presented. The unit cell design incorporates multiple resonant modes to achieve a wide bandwidth and PIN diodes to enable phase control. The unit cell is characterized by two solutions of polarization hold and polarization shifting, and has a compact size of 2.91 × 2.91 mm. Both polarization solutions demonstrate a bandwidth of 29.4–40 GHz (30.5%) at PBC, with perfect 180° phase control. In finite-size environment, the proposed PM also shows good performance. The performance of the proposed PM unit cell is validated through experimental testing in the WR28 waveguide simulator. As shown in Table 2, recently demonstrated wideband 1-bit phase modulating unit cells have utilized strategies like air-gap coupling or multilayer structures to achieve increased bandwidths. However, these approaches increase fabrication complexity and cost. Air-gap coupling also makes the metasurface more fragile and sensitive to damage. At higher frequencies, tiny errors in dimensions and alignment can greatly degrade performance. In contrast, our proposed design achieves wide bandwidth with a simple 4-layer PCB structure. By avoiding air-gaps and minimizing the number of layers, our unit cell is easier to fabricate and assemble. This helps enable scalable large-area metasurfaces. Additionally, the unit cell period of 0.37 ${\lambda }_{0}$ enabling finer spatial sampling for given operating frequencies. The multi-resonance wideband approach provides a promising route towards practical wideband phase modulation metasurfaces.

**Table 2:** Comparison of the performance of wideband 1-bit PM unit-cells.

References	Frequency (GHz)	BW (%)	Thickness (mm)	Layers	Unit cell period	Air-coupled or not
^15^	5	45	10	4	0.29 ${\lambda }_{0}$	Y
^16^	12.5	32	4.66	7	0.48 ${\lambda }_{0}$	N
^18^	11.5	26	4.14	4	0.57 ${\lambda }_{0}$	Y
^20^	17.1	16.4	3.286	5	0.46 ${\lambda }_{0}$	N
This work	34.7	30.5	1.116	4	0.37 ${\lambda }_{0}$	N

## Data Availability

The datasets used and/or analysed during the current study available from the corresponding author on reasonable request.
